# SARS-CoV-2 molecular testing in Greek hospital paediatric departments: a nationwide study

**DOI:** 10.1017/S0950268821000455

**Published:** 2021-02-24

**Authors:** Athanasios Michos, Parthena Savvidou, Garyfallia Syridou, Eirini Eleftheriou, Elias Iosifidis, Ioanna Grivea, Vana Spoulou, Emmanouil Galanakis, George Syrogiannopoulos, Maria Tsolia, Emmanuel Roilides, Vassiliki Papaevangelou

**Affiliations:** 1 1st Department of Pediatrics, School of Medicine, National and Kapodistrian University of Athens, Athens, Greece; 2 3rd Department of Pediatrics, Aristotle University School of Health Sciences, Hippokration General Hospital, Thessaloniki, Greece; 3 3rd Department of Pediatrics, School of Medicine, National and Kapodistrian University of Athens, Athens, Greece; 4 2nd Department of Pediatrics, School of Medicine, National and Kapodistrian University of Athens, Athens, Greece; 5 Department of Pediatrics, School of Medicine, University of Thessaly, Larissa, Greece; 6 Department of Pediatrics, School of Medicine, University of Crete, Heraklion, Greece

**Keywords:** Children, COVID-19, diagnosis, epidemiology, SARS-CoV-2, testing

## Abstract

As most children infected with severe acute respiratory syndrome coronavirus 2 (SARS-CoV-2) present with mild symptoms or they are asymptomatic, the optimal strategy for molecular testing it is not well defined. The aim of the study was to determine the extent and aetiology of molecular testing for SARS-CoV-2 in Greek paediatric departments during the first phase of the pandemic and identify possible differences in incidence, depending on the age group and geographical area. We conducted a nationwide study of molecular testing for SARS-CoV-2 of children in paediatric departments between March and June 2020. A total of 65 paediatric departments participated in the study, representing 4901 children who were tested for SARS-CoV-2 and 90 (1.8%) were positive. Most paediatric cases were associated with topical outbreaks. Adolescents 11–16 years had the highest positivity rate (3.6%) followed by children 6–10 years (1.9%). However, since the testing rate significantly differed between age groups, the modified incidence of SARS-CoV-2 infection per age group was highest in infants <1 year (19.25/10^5^ population). Most children tested presented with fever (70.9%), respiratory (50.1%) or gastrointestinal symptoms (28.1%). Significant differences were detected between public and private hospitals regarding the positivity rate (2.34% *vs.* 0.39%, *P*-value <0.001). Significant variation in SARS-CoV-2 molecular testing positivity rate and incidence between age groups indicate discrepancies in risk factors among different age groups that shall be considered when ordering molecular testing.

## Introduction

Since December 2019, severe acute respiratory syndrome coronavirus 2 (SARS-CoV-2) has rapidly spread around the world causing a major pandemic. Differences in the epidemiology of coronavirus disease 2019 (COVID-19) between countries and age groups have been reported [1, 2]. Although paediatric COVID-19 is usually asymptomatic or mild, there is heterogeneity of clinical presentation and cases of severe disease requiring intensive care unit (ICU) hospitalisation or children developing multisystem inflammatory syndrome have been described [2–4].

Although molecular testing for SARS-CoV-2 is the mainstay for specific diagnosis, health care systems are facing obstacles to organise, as the current testing capacity and availability cannot meet the unprecedented global demands for rapid, reliable and widely accessible molecular diagnosis [5]. Most published data on molecular testing of children for SARS-CoV-2 infection originate from China and limited data have been published from European countries [1, 6].

The optimal strategy for molecular testing in children to avoid transmission in families and in schools has not been well defined. The aim of the study was to determine the extent and aetiology of molecular testing for SARS-CoV-2 in Greek paediatric departments during the first phase of the pandemic and identify possible differences in incidence, depending on the age group and geographical area.

## Patients and methods

A retrospective national multicentre observational cohort study of molecular testing for SARS-CoV-2 in paediatric patients was designed and conducted from the Hellenic Society for Pediatric Infectious Diseases (HELLESPID). An electronic questionnaire (available at: https://forms.gle/HsKLZVmgNXvNRCGp9) was sent in June 2020 to the two children's hospitals and all Greek public and private hospitals having paediatric departments regarding molecular SARS-CoV-2 testing of children and adolescents 0–16 years of age during the first 3 months of the pandemic (1st March–31st May 2020). Initial contact was either via e-mail or phone call, and one participant from each hospital was asked to fill in the questionnaire. The questionnaire included epidemiological data regarding molecular testing for SARS-CοV-2, accessibility to SARS-CοV-2 molecular testing and turnaround time for laboratory diagnosis. Any child with at least one nasal/pharyngeal swab specimen positive for SARS-CoV-2 as tested by real-time reverse-transcriptase polymerase chain reaction (RT-PCR) was defined as SARS-CoV-2 infection.

A second more detailed questionnaire including demographic data and clinical information as well as the reason for which the test was performed was sent to large public paediatric departments of Athens (*n* = 3) and Thessaloniki (*n* = 1). Calculation of modified incidence of SARS-CoV-2 infection per prefecture and per age group was estimated based on the age stratification data given by the Hellenic Statistical authority. In the modified incidence, only positive cases diagnosed in the hospitals were included in the calculation of the nominator of incidence while cases diagnosed through tracing of index cases were excluded.

## Results

**Fig. 1.**
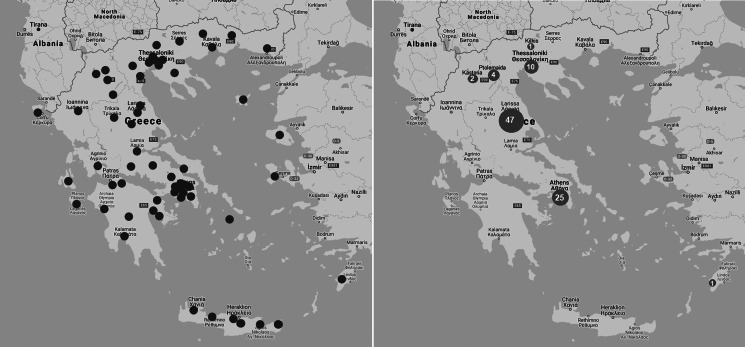
Geographical distribution of the paediatric departments (*n* = 65) which participated in the national Greek paediatric study (panel A) and distribution of children who tested positive for SARS-CoV-2 with RT-PCR (panel B) from 1st March to 31st May 2020.

From the 73 hospitals with paediatric departments asked to participate, 65 (89%) responded. Of them, 60 were public and five were private institutions. The geographical distribution of participating hospitals is shown in Figure 1 (panel A). A total of 4901 children were tested for SARS-CoV-2 and 90 (1.8%) were positive. Most paediatric cases were associated with local outbreaks of SARS-CoV-2 infection in the adult population (Fig. 1, panel B). Significant variation of SARS-CoV-2 positivity rate by month was noted during the study period. More specifically in March 17/990 (1.7%) were tested positive, in April 42/1580 (2.7%) and in May 31/2331 (1.3%) (*P*-value: 0.008, chi-square test).

Significant differences in the positivity rate were also detected among different age groups (Table 1) (*P*-value: <0.0001). Adolescents of 11–16 years had the highest positivity rate (3.6%) followed by children of 6–10 years (1.9%), while children of 1–5 years had the lowest positivity rate (0.9%) (Table 1). Adolescents had a significantly higher relative risk (RR) for positive SARS-CoV-2 RT-PCR result when compared to younger children (RR = 2.62; 95% CI 1.73–3.97). However, since the testing rate significantly differed between age groups, the incidence of SARS-CoV-2 infection per age group was found highest in infants <1 year (Table 1).

**Table 1.** Number of children tested for SARS-CoV-2 with RT-PCR and confirmed infections per age group, Greece from 1st March to 31st May 2020

Age (years)	Tested (*N*)	Positive (*n*)	Positivity rate (%)	Testing rate (per 10^5^ children of the general population)	Modified incidence^a^ (per 10^5^ children of the general population)
<1	1200	20	1.7	1155	19.25
1–5	1706	15	0.9	317	2.79
6–10	966	18	1.9	187	3.49
11–16	1029	37	3.6	164	5.9
Total	4901	90	1.8	275	5.37

a Modified incidence was calculated based only on the index cases of SARS-CoV-2 in each age group.

The mean turnaround time for the SARS-CoV-2 RT-PCR result was 25.13 (s.d.: 17.47) h; in 21% of the hospitals (37/141 responses or 14/65 clinics) molecular testing was available within the facility. However, significant differences were detected between tertiary care and secondary care hospitals regarding turnaround time for the result (mean ± s.d.: 16.5 ± 11.34 *vs.* 30.94 ± 18.28 h, respectively, *P*-value: <0.0001). Significant differences were also detected between public and private hospitals regarding time to result (mean ± s.d.: 26.7 ± 17.57 *vs.* 13.4 ± 9.17 h respectively, *P*-value: 0.002), as well as regarding positivity rate (2.34%, 85/3620 *vs.* 0.39%, 5/1281, respectively, *P*-value: <0.001).

Among 90 children tested positive for SARS-CoV-2, 75 (83%) were hospitalised, while 1 (1.3%) toddler with spinal muscular atrophy required intensive care. Of them, 47 children represent a cluster from a Roma camp in Central Greece, who were isolated in a clinic, to prevent transmission to other family members. There was no death or SARS-CoV-2-associated multisystem inflammatory syndrome recorded.

A more detailed analysis included 1598 children, who were tested for SARS-CoV-2 in tertiary care hospitals in Athens and Thessaloniki and the reason for testing are presented in Table 2. The median age was 3 years (IQR: 0.7–9 years) and 58.6% of them were boys. Overall, 28 children (1.5%) tested positive; of them 19 had clinical manifestations and had presented with fever (65%), and/or respiratory symptoms (55%) and/or gastrointestinal symptoms (10%). No complications were reported in the hospitalised children with COVID-19, and almost all recovered with supportive care. Exception was one toddler with spinal muscular atrophy who was intubated and admitted to ICU, leading to respiratory insufficiency and tracheostomy, who was administered hydroxychloroquine, oseltamivir and corticosteroids. Six neonates of SARS-CoV-2 positive mothers were included in this subpopulation, all of whom tested negative.

**Table 2.** Aetiology for SARS-CoV-2 molecular testing in tertiary care paediatric departments in Athens (*n* = 3) and Thessaloniki (*n* = 1), 1st March—31st May 2020 (*n* = 1598)

Month, *n* (%)	Fever, *n* (%)	Respiratory symptoms, *n* (%)	Gastrointestinal symptoms, *n* (%)	High risk population, *n* (%)	Testing before surgery, *n* (%)	Contact with SARS-CoV-2 positive, *n* (%)
March 264 (16.5)	199 (75)	180 (68.1)	43 (16.2)	9 (3.4)	6 (2.2)	7 (2.6)
April 463 (28.9)	320 (69)	261 (56.3)	158 (34.1)	14 (3)	34 (7.3)	26 (5.6)
May 871 (54.5)	614 (70.5)	360 (41.3)	248 (28.4)	21 (2.4)	129 (14.8)	34 (3.9)
Total (*n* = 1598)	1133 (70.9)	801 (50.1)	449 (28.1)	44 (2.8)	169 (10.6)	67 (4.2)

Several children had more than one reasons for testing.

## Discussion

In the current study, we detected a positivity rate for SARS-CoV-2 (1.8%) lower than the general population positivity rate (2.6%) in Greece during the same period [7]. Nevertheless, most SARS-CoV-2 paediatric cases were detected in specific geographical regions associated with local outbreaks. In the first phase of the pandemic, Greece reported lower incidence of COVID-19 when compared to most other European countries [8]. A possible explanation for the country's low incidence could be that early after the first SARS-CoV-2 adult case in Greece (25th February 2020), school closure (10th March) and community lock-down (16th March) were implemented. Whether school closure affected COVID-19 epidemic curve still remains controversial and challenging for the upcoming months, since the second phase of the epidemic is evolving [9–12].

Similarly to our study, even in countries with high incidence of COVID-19, children were less affected [6, 13, 14]. This is probably due to the fact that children with SARS-CoV-2 infection are usually asymptomatic or have mild symptoms that do not require SARS-CoV-2 testing or hospitalisation [14–16]. Thus, one may postulate that children presenting to the emergency department with mild symptoms were sent home without testing for SARS-CoV-2 or may have not visited healthcare centres at all. Consistent with recently published studies from other countries, only a small percentage of children were confirmed by RT-PCR and even smaller required hospital admission [1, 14, 17].

The strategy of testing only symptomatic children presenting to the hospital has recently been questioned based on a multicentre prospective French study [18]. A symptom-based SARS-CoV-2 testing strategy failed to identify 45% (95% CI 24–68) of hospitalised children infected by SARS-CoV-2. Based on these results authors recommended, in order to limit intra-hospital transmission, to consider a systematic screening of children admitted to hospital [18].

In our population, a higher testing and positivity rate of SARS-CoV-2 was detected in infants <1 year comparing with other age groups. All positive SARS-CoV-2 infants were associated with a positive family contact [19]. This was also found in other studies representing either more symptomatic disease in this age group, higher tendency of families to seek medical advice or higher propensity of clinicians to admit them to hospitals [13, 17]. In contrast, all six infants born from SARS-CoV-2 positive mothers were tested negative for the virus, as strict precautions were taken during labour. Although congenital infection with SARS-CoV-2 is not usual, there are case-reports of possible vertical transmission [20, 21].

A limitation of the study is its retrospective nature; however, its major advantage is that it is presents nationwide data from a region that successfully coped with the SARS-CoV-2 virus during the initial phase of the pandemic. In addition, although no significant complications or adverse events were reported to children with COVID-19 in our population, the low number of infected children does not allow drawing certain inferences.

In conclusion, criteria for molecular testing in paediatric population in the first phase of SARS-CoV-2 pandemic included a variety of symptoms from upper and lower respiratory or gastrointestinal tract or asymptomatic tracing. However, significant variation in SARS-CoV-2 molecular testing positivity rate and incidence between age groups indicate discrepancies in clinical manifestations or risk factors among different age groups that shall be considered when ordering molecular testing. Prospective surveillance studies are essential to identify specific risk factors associated with specific age groups and optimise testing and management strategies in children.

## Data Availability

Data included in this paper are available upon request to the corresponding author.
